# Heterogeneity in subjective cognitive decline in the Sino Longitudinal Study on Cognitive Decline(SILCODE): Empirically derived subtypes, structural and functional verification

**DOI:** 10.1111/cns.14327

**Published:** 2023-07-20

**Authors:** Zhenrong Fu, Mingyan Zhao, Yuxia Li, Yirong He, Xuetong Wang, Zongkui Zhou, Ying Han, Shuyu Li

**Affiliations:** ^1^ Key Laboratory of Adolescent Cyberpsychology and Behavior (CCNU) Ministry of Education Wuhan China; ^2^ School of Psychology, Key Laboratory of Human Development and Mental Health of Hubei Province Central China Normal University Wuhan China; ^3^ Department of Psychology Tangshan Gongren Hospital Tangshan China; ^4^ Department of Neurology Tangshan Central Hospital Tangshan China; ^5^ Department of Neurology Xuanwu Hospital of Capital Medical University Beijing China; ^6^ State Key Laboratory of Cognitive Neuroscience and Learning Beijing Normal University Beijing China; ^7^ Key Laboratory of Biomedical Engineering of Hainan Province, School of Biomedical Engineering Hainan University Haikou China; ^8^ Center of Alzheimer's Disease Beijing Institute for Brain Disorders Beijing China; ^9^ National Clinical Research Center for Geriatric Disorders Beijing China; ^10^ Institute of Biomedical Engineering Shenzhen Bay Laboratory Shenzhen China

**Keywords:** Alzheimer's disease, heterogeneity, neuroimaging, neuropsychology, subjective cognitive decline

## Abstract

**Aims:**

We evaluated whether Subjective Cognitive Decline (SCD) subtypes could be empirically derived within the Sino Longitudinal Study on Cognitive Decline (SILCODE) SCD cohort and examined associated neuroimaging markers, biomarkers, and clinical outcomes.

**Methods:**

A cluster analysis was performed on eight neuropsychological test scores from 124 SCD SILCODE participants and 57 normal control (NC) subjects. Structural and functional neuroimaging indices were used to evaluate the SCD subgroups.

**Results:**

Four subtypes emerged: (1) dysexecutive/mixed SCD (*n* = 23), (2) neuropsychiatric SCD (*n* = 24), (3) amnestic SCD (*n* = 22), and (4) cluster‐derived normal (*n* = 55) who exhibited normal performance in neuropsychological tests. Compared with the NC group, each subgroup showed distinct patterns in gray matter (GM) volume and the amplitude of low‐frequency fluctuations (ALFF). Lower fractional anisotropy (FA) values were only found in the neuropsychiatric SCD group relative to NC.

**Conclusion:**

The identification of empirically derived SCD subtypes demonstrates the presence of heterogeneity in SCD neuropsychological profiles. The cluster‐derived normal group may represent the majority of SCD individuals who do not show progressive cognitive decline; the dysexecutive/mixed SCD and amnestic SCD might represent high‐risk groups with progressing cognitive decline; and finally, the neuropsychiatric SCD may represent a new topic in SCD research.

## Graphical abstract

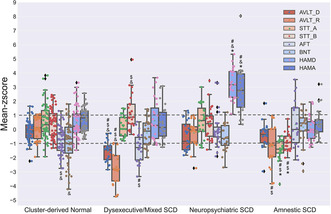

## 1 INTRODUCTION

Individuals with subjective cognitive decline (SCD) are characterized by persistent self‐experienced cognitive decline without explicit performance issues on neuropsychological assessment.^1^ Accumulated evidence indicates that SCD is a putative precursor to mild cognitive impairment (MCI) or dementia.^2^, ^3^ However, previous research observed inconsistent and heterogeneous neuroimaging findings in individuals with SCD,^4^, ^5^ which demonstrates the underlying heterogeneity of pathology may exist in the SCD population.

Recent progress in clinical, neuroimaging and pathological research found significant heterogeneity in MCI and Alzheimer's disease (AD) populations.^6^, ^7^, ^8^ Exploring this heterogeneity may help advance our understanding of the different neuropathological mechanisms associated with AD. However, earlier research on SCD has mainly focused on determining the quantitative and qualitative aspects of SCD in relation to underlying AD pathology.^5^, ^9^, ^10^ In contrast, despite being a high‐risk population for future dementia, the characterization of heterogeneity within individuals with SCD has been largely neglected. Recent studies have revealed that, depending on the cognitive domain being impaired, different associations can be observed between biomarkers, network indices, and cognitive performance in individuals with SCD,^11^, ^12^ which calls for a more in‐depth investigation in the diversity of neuropathological underpinnings of SCD.

The neuropsychological performance is primarily used to distinguish SCD from the MCI stage.^2^ Currently, cognitive scores have been widely utilized in diagnosing and categorizing AD‐related patients.^13^ To explore the heterogeneity of MCI in a nonbiased manner, several studies have applied cluster‐analytic techniques on neuropsychological profiles rather than relying on prespecified theoretical cut‐points in one or more cognitive domains.^13^, ^14^ For instance, Delano‐Wood et al. provided one of the first pieces of evidence by clustering 70 MCI patients into three distinct groups with different white matter (WM) lesion types.^15^ Similarly, Edmonds et al.^6^ and Machulda et al.^13^ also performed cluster analyses on MCI patients from Alzheimer's Disease Neuroimaging Initiative (ADNI) and the Mayo Clinic Study of Aging (MCSA) cohort, respectively, which identified four subtypes: dysnomic, dysexecutive, and amnestic, as well as a cluster‐derived normal group. Furthermore, heterogeneous patterns of cortical atrophy, as observed in magnetic resonance imaging (MRI) scans, have been reported in cross‐sectional and longitudinal research of these subtypes.^16^, ^17^ While the evidence is suggestive of the presence of subtypes of MCI which can be empirically identified, there is yet to be any research which explores the heterogeneity of SCD based on neuropsychological profiles and cluster‐analytic techniques, which may have important clinical and diagnostic implications.

Neuropsychiatric symptoms (NPSs) are common in patients with AD, and the type and severity of such symptoms vary across the different stages of AD.^18^, ^19^ Previous studies on SCD have demonstrated subthreshold symptoms of depression and anxiety in the preclinical AD stage.^1^ Notably, the Subjective Cognitive Decline Initiative (SCD‐I) working group suggested that subthreshold symptoms should be taken into consideration in statistical models, as opposed to excluding individuals with such symptoms from studies on SCD.^1^ In addition, various studies^20^, ^21^, ^22^ suggest that depressive symptoms may be a more reliable predictor of subjective cognitive complaints than objective memory performance. Cognitive impairments and NPSs can exist independently and concurrently, and may be underpinned by similar neuropathology while also potentially mutually reinforcing.^23^ Consequently, individuals with subthreshold NPSs in the SCD population should be considered as an essential subtype.

This study endeavors to expand upon the existing empirical research on MCI subtypes by identifying subtypes of SCD based on neuropsychological profiles within the cohort of the Sino Longitudinal Study on Cognitive Decline (SILCODE).^24^ Should any subtypes exist, the current work will examine the associated clinical characteristics, biological markers, and will specify abnormal structural and functional patterns in these SCD subtypes. This study provides novel insights that may enhance the comprehension of the underlying neuropathology of SCD and elucidate the diagnostic significance of SCD in the preclinical stage of AD.

## 2 METHODS

### 2.1 Participants

This study included 181 right‐handed, native Chinese participants, all from the baseline dataset of the SILCODE^24^ from March 20, 2017 to September 17, 2018. This study was performed in accordance with Medical Research Ethics Committee and Institutional Review Board of XuanWu Hospital, and every subject gave their written informed consent to participate. The SILCODE is listed in the ClinicalTrail.gov registry (NCT02225964). The participant inclusion criteria were as follows: (1) 59–79 years old, right‐handed and Mandarin‐speaking subjects; (2) no history of stroke, brain tumors, brain injury, Parkinson's disease, encephalitis, or epilepsy; (3) no history of diseases that could cause cognitive decline (eg, thyroid dysfunction, severe anemia, syphilis, or HIV); (4) Hamilton Anxiety Scale (HAMA) lower than 29 and Hamilton Depression Scale (HAMD) lower than 24; (5) no obvious microvascular disease.

### 2.2 Neuropsychological assessment

Neuropsychological tests measured three cognitive domains including episodic memory (Auditory Verbal Learning Test (AVLT) HuaShan version^25^), language (Animal Fluency Test (AFT), 30‐item Boston Naming Test (BNT),^24^) and attention /executive function (Shape Trail Test (STT) Parts A and B^26^). Global cognition was examined by the Montreal Cognitive Assessment Basic Version (MoCA‐B).^27^ In addition, HAMA and HAMD were used to evaluate the NPSs. Besides, the SCD questionnaire, including nine reliable SCD items (SCD‐9), was used for the quantitative evaluation of SCD severity.^28^

SCD participants were collected according to Jessen's criteria^2^: (1) presence of self‐experienced persistent cognitive decline compared with previous normal status and unrelated to an acute event; (2) concerns associated with memory complaint or confirmation of cognitive decline by an observer; (3) failure to meet the criteria for MCI^29^ or dementia. The normal control (NC) participants were within the normal range upon every cognitive test and had no confirmed subjective cognitive complaint. Finally, 57 NC and 124 SCD individuals were included in this study.

### 2.3 Imaging acquisition and processing

#### 2.3.1 MRI data acquisition and processing

MRI data in the study were acquired using an integrated simultaneous 3.0 T TOF PET/MR (SIGNA PET/MR, GE Healthcare, Milwaukee, Wisconsin, USA) at the Xuanwu Hospital of Capital Medical University. See Appendix S1 for details. Finally, 124 SCD individuals and 57 NC individuals were included in the voxel‐based morphometry (VBM) analysis; 121 SCD individuals and 55 NC individuals were included in the voxel‐wise amplitude of low‐frequency fluctuations (ALFF) analysis; 123 SCD individuals and 56 NC individuals were included in the tract‐based spatial statistics (TBSS) analysis.

### 2.4 Statistical analyses

The cluster groups were derived based on two language measures, two attention/executive function measures, two memory measures, and two neuropsychiatric measures. First, raw neuropsychological scores for each SCD participant were transformed into age‐, gender‐ and education‐adjusted z scores based on regression coefficients derived from the NC group. Second, a hierarchical cluster analysis was performed on the z scores with Ward's method, consistent with previous MCI studies.^6^, ^15^ Third, a discriminant function analysis (DFA) was conducted to quantitatively examine the ability of the eight neuropsychological scores to discriminate the cluster subgroups. Following the above steps, all SCD individuals were classified into four subtypes.

Shapiro–Wilk test for normality was used to assess data distribution. Kruskal‐Wallis H test, analysis of variance and Chi‐square test were used to examine subgroup differences in demographic, neuropsychological and biomarker features (Bonferroni corrected). A general linear model was performed with age, sex, years of education, and total intracranial volume (TIV) (hippocampal subfields use hippocampal volume) as covariates to determine the between‐group differences of volume and cortical thickness (Bonferroni corrected).

Voxel‐wise general linear model analyses were used to examine between‐group differences in ALFF^30^ and GM volume. The covariates in VBM analysis were age, gender, years of education, and TIV, and in voxel‐based ALFF analysis were age, gender, and years of education. Specific T contrasts were established to map the significant differences in voxel‐wise GM and ALFF values between subgroups and the NC group. Regarding the VBM analysis, resulting maps were obtained using two‐tailed Gaussian random field (GRF)^31^ (voxel: *p* < 0.005 and cluster: *p* < 0.01) correction and cluster sizes larger than 337.5 mm^3^ were reported. Regarding voxel‐wise ALFF analysis, resulting maps were obtained using two‐tailed GRF (voxel: *p* < 0.005 and cluster: *p* < 0.05) correction and cluster sizes larger than 540 mm^3^ were reported. The coordinates of the peak intensity of the cluster within the scope of the automated anatomical labeling 3 (AAL3) atlas^32^ or Brodmann area (BA) were reported. Regarding TBSS analysis, we compared FA in WM between participants in subgroups and the NC group using non‐parametric permutation‐based testing (age, gender, years of education, and image resolution as covariates). Significant differences were estimated by 5000 random permutations using threshold‐free cluster enhancements (TFCE) and family‐wise error (FWE) corrections (*p* < 0.05). The John Hopkins University (JHU) WM tractography atlas and JHU‐ICBM‐DTI‐81 WM labels atlas^33^ were used to identify regions of statistical significance.

We use a partial correlation model (corrected for age, gender, and education) to determine the relationship between GM volume (or ALFF or FA) and clinical performance. Correlation analyses were separately performed in each subgroup and considered significant at *p* < 0.05 (Bonferroni corrected). The GM volume and ALFF values were represented by mean values of the significant different peak regions extracted by spheres with a radius of 6 mm around the peak intensity coordinates. The FA values in WM were represented by the mean values of the significant clusters.

Finally, a general linear model was used to determine the specificity of neuroimaging indices (GM volume, ALFF values, and FA values) above across all sub groups, extracted from the significantly different peak regions (age, gender, and education as covariates).

## 3 RESULTS

### 3.1 Cluster analysis and DFA

**FIGURE. 1:**
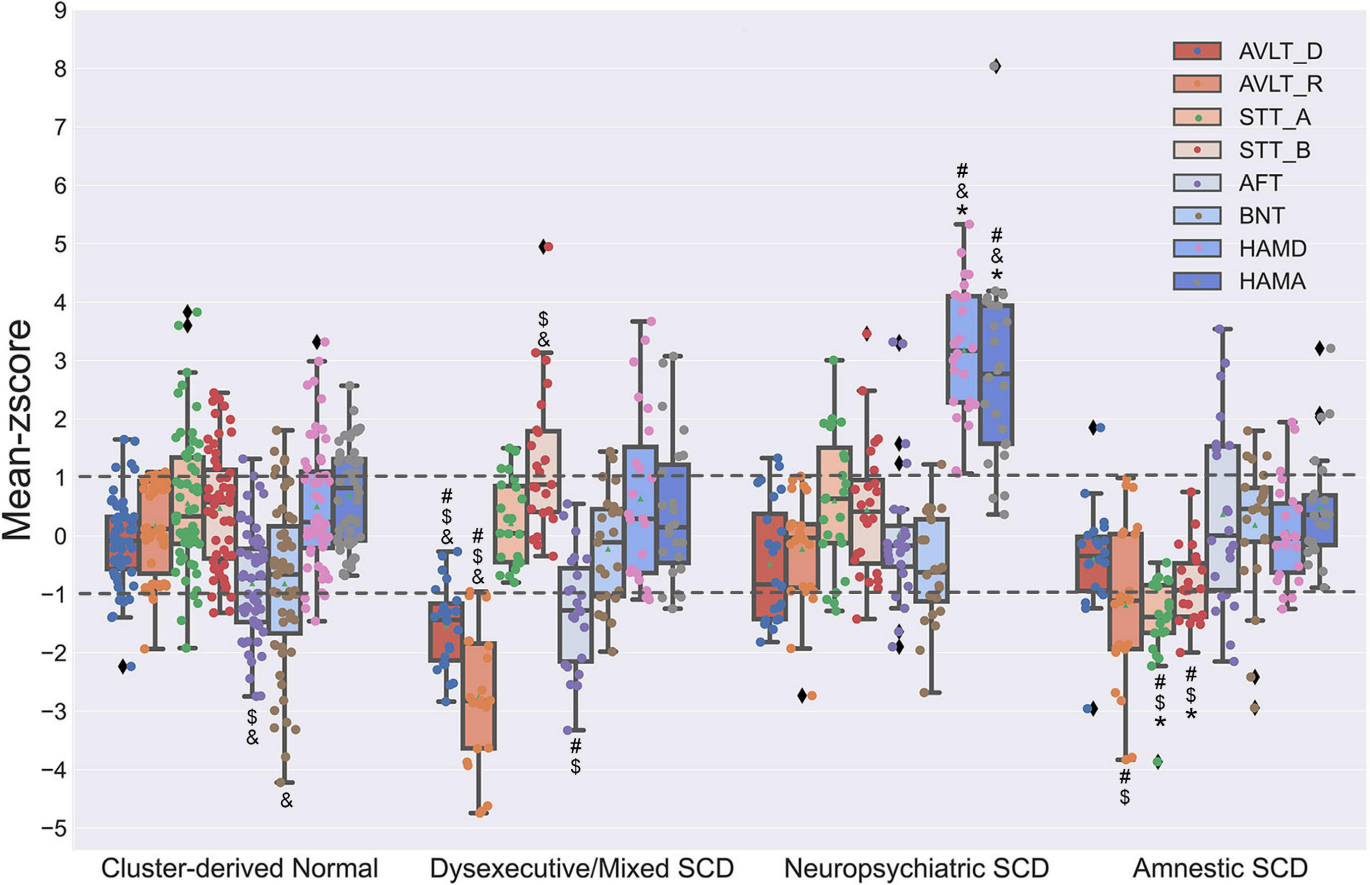
Neuropsychological performance for the cluster groups. Error bars denote standard deviations (SDs). The horizontal dotted line indicates the cut‐point for impairment (1 SDs). AFT, Animal Fluency Test; AVLT_D, Auditory Verbal Learning Test long delayed memory; AVLT_R, AVLT recognition; BNT, 30‐item Boston Naming Test; HAMA, Hamilton Anxiety Scale; HAMD, Hamilton Depression Scale; STT_A, Shape Trail Test part A; SCD, Subjective Cognitive Decline; STT_B, STT part B. #, showed significant difference with cluster‐derived normal group; *, showed significant difference with dysexecutive/mixed SCD group; $, showed significant difference with neuropsychiatric SCD group; &, showed significant difference with amnestic SCD group.

A cluster analysis of the neuropsychological profiles from 124 SCD individuals resulted in four distinct subgroups based on the mean performance for each group (see Figure 1): (1) cluster‐derived normal group (*n* = 55; 44.35%), performed within the normal limits on neuropsychological tests; (2) dysexecutive/mixed SCD group (*n* = 23; 18.55%), showed a significant deficit in executive function, as well as impairments in memory and naming; (3) neuropsychiatric SCD (*n* = 24;19.36%), with isolated subthreshold symptoms in depression and anxiety and performed normally in cognitive tests; (4) amnestic SCD (*n* = 22; 17.74%), with cognitive impairment isolated in memory.

We performed a linear discriminant analysis which can accurately classify 96.8% of participants, and cross‐validation using the leave‐one‐out method showed only a mild expected reduction in correct classification (91.9%). A four‐cluster solution was optimal relative to a three‐cluster solution that combined the dysexecutive/mixed and amnestic groups into one group (as the decline in memory is a crucial inclusion criterion for SCD, and amnestic subtype is a traditional MCI subtype). Due to this study only including 124 SCD participants, a five‐cluster solution may produce unbalanced groups with few subjects.

### 3.2 Clinical and biomarker characteristics of SCD subtypes and NC group

No differences were found in age, gender, and years of education across the five groups (all *p* > 0.05) (see Table 1). There were significant group differences on all eight neuropsychological measures (*p* ≤ 0.001) (see Table 1). Post hoc tests with Bonferroni correction determined that: (1) the dysexecutive/mixed SCD group performed worse than all other groups on AVLT long‐ delayed memory (all *p* ≤ 0.008); (2) the dysexecutive/mixed SCD group performed worse than all other groups on one measure of memory (AVLT recognition) (all *p* ≤ 0.05), and the amnestic SCD group performed worse than cluster‐derived normal group and NC group (all *p* ≤ 0.01); (3) the amnestic SCD group performed better (*p* < 0.001) than all other groups on one execution/attention measure (STT part A); (4) regarding the STT test part B, the amnestic SCD group performed better than all other groups (*p* ≤ 0.002), and the dysexecutive/mixed SCD group performed worse than NC group (*p* = 0.002); (5) regarding the AFT test, the cluster‐derived normal and the dysexecutive/mixed SCD group performed worse than amnestic SCD group and NC group (all *p* ≤ 0.011), and the dysexecutive/mixed SCD group performed worse than the neuropsychiatric SCD group (*p* = 0.005); (6) regarding the BNT test, the cluster‐derived normal performed worse than amnestic SCD and NC group (all *p* ≤ 0.024); (7) regarding the HAMA scale, the neuropsychiatric SCD group performed worse than all other groups (*p* < 0.001), and the NC group performed better than the cluster‐derived normal group (*p* = 0.002); (8) the neuropsychiatric SCD group performed worse than all other groups (*p* < 0.001) on the HAMD scale. Noted that the higher the score of the memory and naming tests, the better the performance. On the contrary, the higher the score of the execution and neuropsychiatric tests, the worse the performance.

**TABLE 1:** Demographic, neuropsychological and biomarker characteristics of the cluster groups and normal control group.

Variable	Cluster‐derived Normal	Dysexecutive/mixed SCD	Neuropsychiatric SCD	Amnestic SCD	Normal control	Statistic value	*p* value
Age (years)	65.58 (4.85)	67.22 (4.46)	65.71 (4.33)	65.95 (4.73)	66.63 (4.52)	4.441^b^	0.35
Gender (M/F)	15/40	12/11	4/20	8/14	21/36	8.009^c^	0.091
Education (years)	12.22 (3.19)	12.78 (2.89)	12.38 (2.67)	13.39 (2.24)	12.42 (2.96)	2.978^b^	0.562
AVLT long delayed memory	7.76 (1.54)	5 (1.21)	7 (2)	7.32 (1.86)	7.98 (2.03)	41.85^b^	<0.001
AVLT recognition	23.16 (0.88)	20.09 (1.28)	22.75 (0.99)	21.77 (1.48)	22.98 (1.06)	64.121^b^	<0.001
STT part A(s)	64.04 (14.91)	60.91 (9.58)	64.61 (15.09)	39.64 (8.74)	57.97 (14.96)	15.579^d^	<0.001
STT part B(s)	140.2 (27.65)	160.17 (33.44)	139.29 (33)	103.5 (15.49)	128.53 (26.94)	43.235^c^	<0.001
AFT	17.73 (3.34)	16.26 (3.48)	20.67 (4.4)	21.91 (5.61)	20.61 (3.48)	33.306^b^	<0.001
BNT	24.36 (3.08)	25.61 (2.02)	24.88 (2.05)	26.55 (2.52)	26.09 (2.14)	16.705^b^	0.002
HAMA	4.38 (2.23)	3.74 (3.24)	10.08 (4.5)	3.86 (2.57)	2.6 (2.71)	56.220^b^	<0.001
HAMD	3.47 (2.8)	3.87 (3.68)	10.58 (3.01)	2.55 (2.2)	2.14 (2.73)	60.789^b^	<0.001
MoCA‐B	25.93 (2.14)	24.09 (1.86)	25.58 (2.02)	26.82 (1.87)	27.04 (1.88)	32.905^b^	<0.001
SCD‐9	4.88 (1.53)	5.07 (1.56)	5.98 (1.75)	5.3 (1.88)	3.61 (2.32)	26.072^b^	<0.001
APOE ε4 carriers (%)^a^	30.2	30.43	25	27.27	12.28	6.136^c^	0.189
TIV (10^6^ mm^3^)	1.396 (0.126)	1.438 (0.132)	1.39 (0.132)	1.443 (0.167)	1.446 (0.142)	5.326^b^	0.255

Abbreviations: AFT, Animal Fluency Test; AVLT, Auditory Verbal Learning Test; BNT, 30‐item Boston Naming Test; F, female; HAMA, Hamilton Anxiety Scale; HAMD, Hamilton Depression Scale; M, male; MoCA‐B, Montreal Cognitive Assessment‐Basic; SCD, Subjective Cognitive Decline; STT, Shape Trail Test; TIV, Total Intracranial Volume.

^a^ Number of participants for APOE analysis: Cluster‐derived Normal: *n* = 53, Dysexecutive/mixed SCD: *n* = 23, Neuropsychiatric SCD: *n* = 24, Amnestic SCD: *n* = 22, Normal control: *n* = 57.

^b^ Kruskal‐Wallis H test.

^c^ Chi‐square test.

^d^ Analysis of variance.

Significant differences were shown in MoCA and SCD‐9 across the five groups (all *p* < 0.001). The dysexecutive/mixed SCD group performed worse than all other groups except the neuropsychiatric SCD group (all *p* ≤ 0.007) on the global cognition measure (MoCA), and the NC group performed better than all other groups except the dysexecutive/mixed SCD group (SCD‐9) (all *p* ≤ 0.043) (see Table 1 and Figure S1). Finally, there were no significant differences in the rate of APOE ε4 carriers across all the four‐cluster groups and the NC group (see Table 1).

### 3.3 Structural and functional patterns relative to NC

**FIGURE 2:**
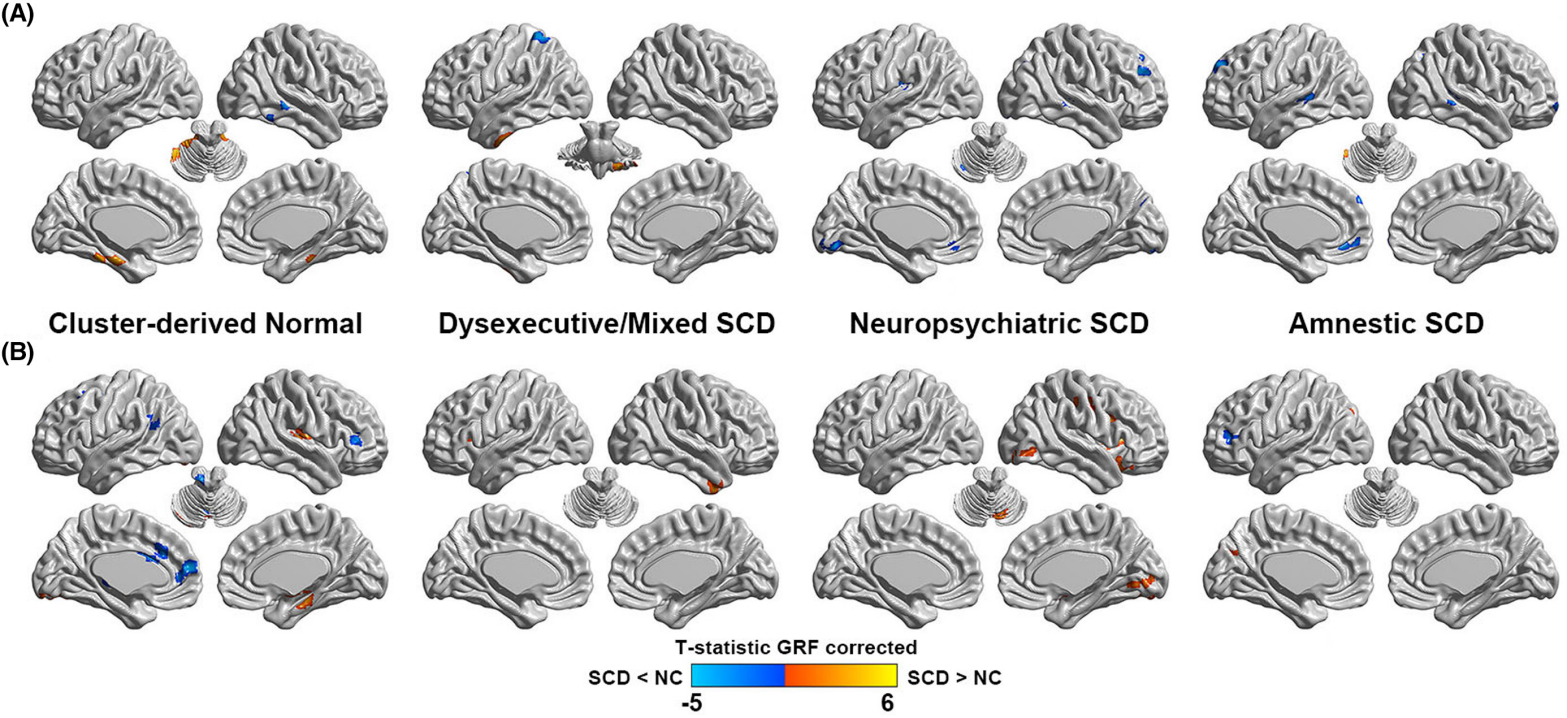
Voxel‐wise GM volume (A) and ALFF values (B) on the left and right lateral and medial pial surfaces for each cluster‐derived group relative to the normal control (NC) group. GRF, Gaussian random field correction; SCD, Subjective Cognitive Decline.

Significant differences in volume between each cluster‐derived SCD subtype relative to the NC are displayed at the voxel‐wise level on the lateral and medial surface view in Figure 2A. Regarding the cluster‐derived normal group, volume reduction was found in the occipital and temporal lobe, and increased volume was shown in the cerebellum regions (see Table S1). Regarding the dysexecutive/mixed SCD group, volume reduction was found in the parietal lobe, while increased GM volume was shown in the temporal lobe and cerebellum regions. Regarding the neuropsychiatric SCD group, volume reduction was found in the rolandic operculum, occipital, frontal, temporal lobes, and cerebellum regions. Regarding the amnestic SCD group, volume reduction was shown in the parietal, frontal, and temporal lobes, and the cerebellum volume increased.

Significant differences in ALFF between cluster‐derived SCD subtype relative to the NC are displayed at the voxel‐wise level on the lateral and medial surface view in Figure 2B. Regarding the cluster‐derived normal group, higher ALFF values were exhibited in temporal lobe and cerebellum regions, and lower values were found in frontal and temporal lobe, as well as cingulate gyrus and cerebellum regions (see Table S2). Regarding the dysexecutive/mixed SCD group, higher ALFF values were exhibited in temporal lobe and insula. Regarding the neuropsychiatric SCD group, lower ALFF values were exhibited in occipital and frontal lobes, as well as precentral gyrus, putamen and cerebellum regions. Regarding the amnestic SCD group, lower values were showed in occipital lobe and higher values were exhibited in frontal lobe.

**FIGURE 3:**
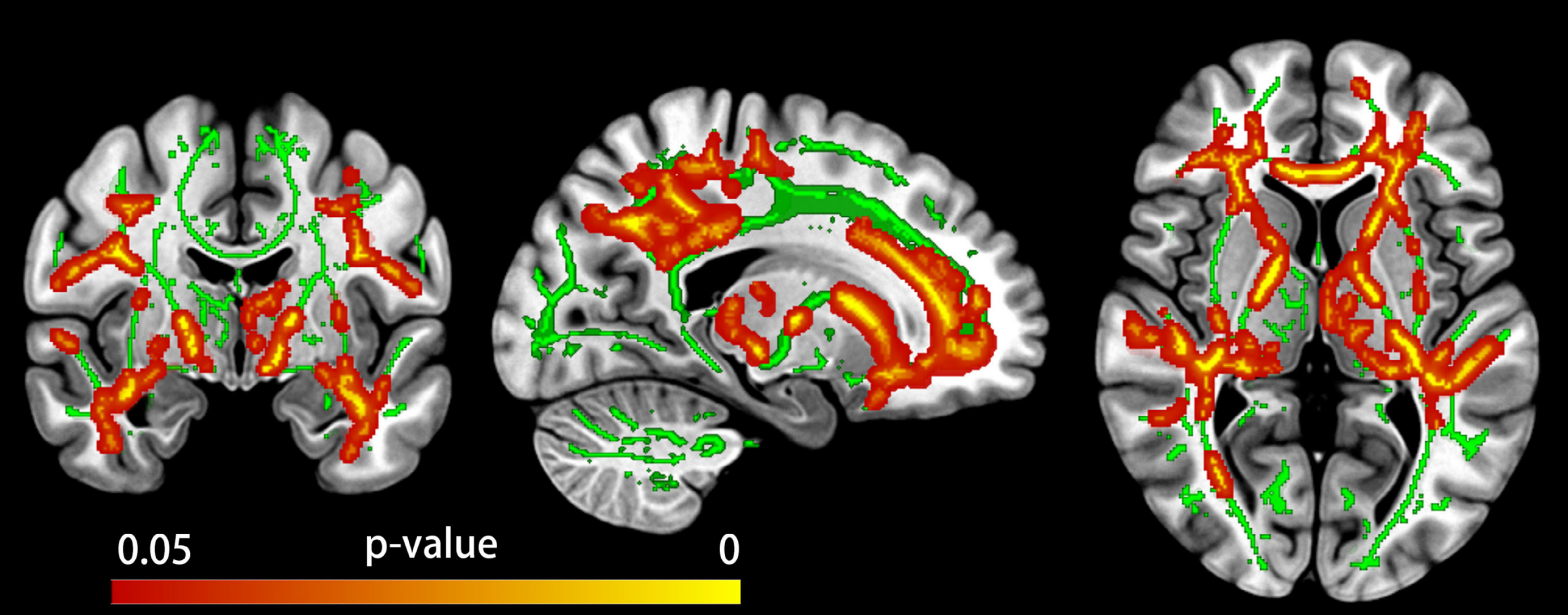
Fractional anisotropy (FA) findings for the neuropsychiatric SCD group relative to the normal control (NC) group. The averaged skeleton (green color) was overlaid with significantly lower FA (red‐yellow color) in the neuropsychiatric SCD group compared with NC group.

After TFCE and FWE correction, significant differences of FA in WM based on TBSS between cluster‐derived SCD subtypes relative to the NC were only found in the neuropsychiatric SCD group (see Figure 3). Compared with the NC group, lower FA values were exhibited mainly in the corpus callosum, corona radiata, superior longitudinal fasciculus, thalamic radiation, and internal capsule in the neuropsychiatric SCD group (see Table S3).

Regarding the regional morphology analysis, the differences of the volume in the hippocampus and three hippocampal subfields and the cortical thickness of the whole brain were compared between cluster‐derived SCD subtypes and NC group. The dysexecutive/mixed SCD group showed a smaller CA1 volume (*p* = 0.027), and the neuropsychiatric SCD group showed a larger hippocampal volume (*p* = 0.018) than the NC group. In addition, the cortical thickness of the left supramarginal was thinner in the amnestic SCD group than in the NC group (*p* = 0.0128) (see Figures S2 and S3).

**FIGURE 4:**
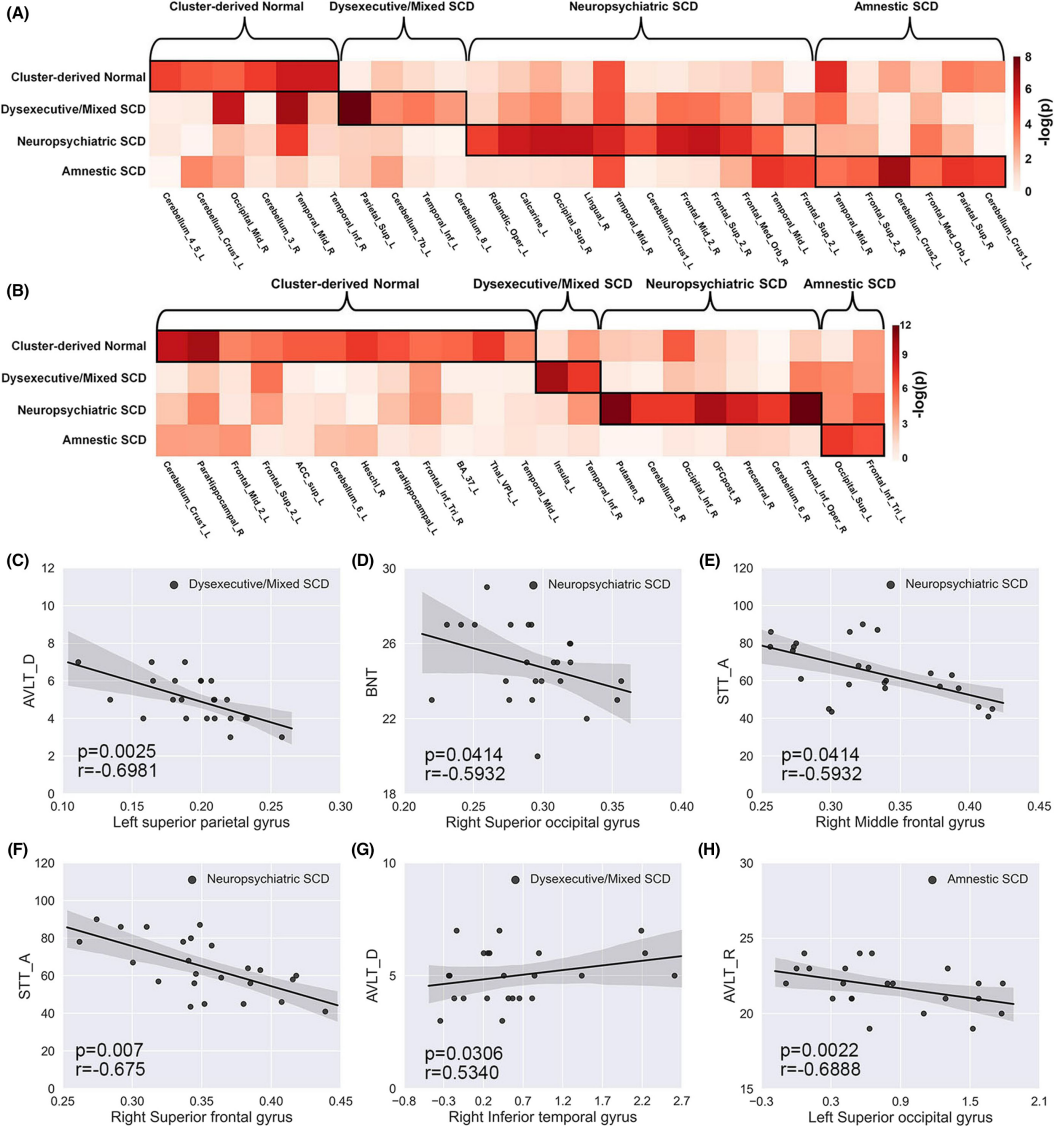
GM volume (A) and ALFF values (B) differ in significant peak regions relative to normal control across all groups and associations between the peak region's GM volume (C–F) or ALFF values (G, H) and cognitive test scores within each subgroup. GM volume and ALFF values differ in significant peak regions relative to normal control across all groups. Every column in the matrix represents the peak region's GM volume or ALFF values. SCD, Subjective Cognitive Decline; L, Left; R, Right; BA, Brodmann area.

In addition, we compared the central effect of the volume and ALFF, which exhibited significant between‐group differences across all groups. As shown in Figure 4, the structural and functional indices showed specificity relative to NC in each subgroup (see Tables S4 and S5).

### 3.4 Associations with neuroimaging indices and clinical performance

The correlations between the significant structural and functional indices and the clinical test scores were separately calculated across subgroups. The peak volume of the significant between‐group regions exhibited a significant correlation with the clinical measures and were illustrated in Figure 4C–F. In addition, the peak ALFF value of the significant between‐group regions also exhibited significant correlations with the clinical measures shown in Figure 4G,H.

## 4 DISCUSSION

We empirically derived subgroups from the SILCODE SCD cohort using cluster analysis based on eight neuropsychological measures and examined patterns of structural and functional indices of each cluster‐derived subgroup relative to NC. Four SCD subgroups emerged: dysexecutive/mixed, neuropsychiatric, amnestic, and a cluster‐derived normal group. The cluster‐derived normal group comprised 44.35% of the SILCODE SCD sample and was comparable with a robust NC group in neuropsychological test performance. In addition, 19.36% of the participants constituted a neuropsychiatric group, exhibiting higher scores on the HAMD and HAMA than other subgroups. Further, the central effects of the significant neuroimaging indices relative to the NC group were found to be specific across all SCD subgroups.

None of the studies explored potential heterogeneity in the SCD population based on neuropsychological profiles and cluster‐analytic techniques. As an expansion and optimal of previous MCI studies,^6^, ^13^, ^14^, ^15^ current results are consistent with previous cluster analytic studies in MCI. The dysexecutive/mixed and amnestic subgroups share features with the subtypes identified in previous MCI^6^, ^13^ and cognitive normal studies.^34^ In addition, the dysexecutive/mixed and amnestic subgroups are classical diagnostic groups no matter in different MCI diagnostic criteria^29^, ^35^ or in cognitive normals.^34^ Similar to a previous MCI study,^6^ the cluster‐derived normal group in SCD may also have a false‐positive error, because most individuals with SCD will not show progressive cognitive decline^2^ and the volunteers in SILCODE are mainly from the community.^36^ As a previous study suggested, individuals recruited from a memory clinic (versus a volunteer/community sample) may have a higher probability of having preclinical AD because they have specific concerns sufficient to prompt a medical visit,^36^ which was proposed as a new feature of SCD plus.^2^ However, differences were found compared with those derived from the ADNI and MCSA MCI cohort, as this study did not include a dysnomic group.^6^, ^13^ These differences may be attributed to the sampling and measuring methods of the SILCODE SCD sample, which is small and unbalanced, and the lack of sensitivity of naming tests in the early stage. Moreover, a prospective study utilizing cut‐points for subtle cognitive impairment in cognitively normal individuals only included executive and memory tests.^34^

It is noteworthy that a new cluster, termed the neuropsychiatric SCD group, was proposed in this study. Various psychiatric disorders can be associated with SCD, and in many studies on SCD in preclinical AD, the participants showed subthreshold symptoms of depression and anxiety.^1^ A previous study proposed that 80% of AD patients show NPSs,^37^ and most NPSs occur in preclinical AD and MCI stages, especially depression and anxiety.^18^ Before reaching the clinical diagnosis criteria, microglial activation and inflammatory signals in AD patients may explain the occurrence of NPSs in the early stages of the AD continuum.^18^, ^38^, ^39^, ^40^ For now, the relationship between NPSs and the underlying pathology of AD is still unclear. Therefore, it is also possible that SCD individuals with NPSs, independent of preclinical AD, may progress to major psychiatric disorders. However, regardless of the relationship between SCD and AD pathology, SCD individuals with NPSs may be an important topic for future conceptualization and research.^1^ The neuropsychiatric SCD group showed lower GM volume and FA values and higher ALFF values than the NC group, which were similar to pervious SCD studies.^5^ Associative fiber tract neurodegeneration in the WM of AD may arise from GM atrophy and Wallerian degeneration.^41^ Noting that lower FA values were only found in the neuropsychiatric SCD group, the result was consistent with previous SCD studies which specify that the SCD group exhibited lower FA values compared with the NC group. Therefore, we speculate that the outcomes indicate that the NPSs may enhance and accelerate AD pathology. In addition, the altered structural and functional indices were commonly located in the prefrontal cortex, a critical damaged region across all NPSs in AD patients.^18^ Expect the neuropsychiatric SCD group, the synchronization of increased and decreased GM volume and ALFF values in different brain regions were found in all other subgroups.

Regarding the dysexecutive/mixed and amnestic SCD subgroups, the cognitive capacity in those groups was impaired (mean z scores lower than 1). The amnestic subgroups performed better than all other groups on execution/attention measure, which is in line with previous MCI studies.^6^, ^15^, ^42^ A previous study proposed that minor neuropsychological deficits in individuals with SCD exhibited moderate association with lower biomarker levels.^12^ Compared with the NC group, the amnestic group exhibited lower GM volume in cortical structures, and the dysexecutive/mixed groups showed higher ALFF values. Interestingly, both an increase and decrease in volume and ALFF were observed in the dysexecutive/mixed and amnestic groups, and the locations of the changes were different. Several potential sources may account for the observed results. First, paradoxically higher regional GM volumes were observed in non‐demented individuals along the AD continuum, which might be due to microglia activation leading to inflammation or leakage of the blood–brain barrier.^43^ Second, higher and lower cerebellar GM volumes have been observed in MCI patients compared with older individuals.^44^ Third, inconsistent results from functional neuroimaging analyses were proposed by various studies.^4^, ^5^ A nonlinear change trajectory over the progression from subjective to objective impairment, and unbalanced subjects in a dataset may be a persuasive explanation.^4^ In previous research, discrepancies have been observed in the imaging signatures of SCD in terms of structure (primarily in the medial temporal lobe) and function (connections between the posterior default mode network and other regions).^5^ These inconsistent findings might be attributed to the fact that functional imaging appears to be more sensitive to AD pathology compared to structural imaging.^30^

Regarding the association between the significant neuroimaging indices relative to NC and neuropsychological tests, significant relationships were found between GM volumes and STT part A scores in the neuropsychiatric SCD group. However, both negative and positive relationships were observed in this study. These inconsistencies are likely related to the heterogeneous nature of SCD groups and the inconsistency of the neuroimaging analyses. Additionally, previous studies for the MCI^15^, ^16^, ^17^ have not calculated the associations between the neuroimaging indices and neuropsychological test scores, potentially because of uncertainty in the outcomes.

Several limitations in the present study should be addressed. First, the SCD subjects in SILCODE is smaller than the MCI subjects in ADNI. Larger sample size may result in more consistent subtypes. Second, we cannot determine if the SCD individuals are in the AD preclinical stage because of insufficient biomarker data, such as the cerebrospinal fluid (CSF) concentrations of hyperphosphorylated tau (p‐tau_181p_) and β‐amyloid (Aβ_1‐42_). Finally, the absence of longitudinal data may restrict our ability to capture the progressive trajectory of the clinical outcomes.

In summary, four specific SCD subtypes were identified in the SILCODE cohort. The cluster‐derived normal group performing within normal limits on all eight neuropsychological measures may represent that most individuals with SCD will not show progressive cognitive decline. Based on a previous study,^12^ we speculated that the dysexecutive/mixed and amnestic SCD groups might represent a higher risk of progressing cognitive decline. However, the evidence looks insufficient without sufficient biomarkers and longitudinal data. A strength of our study is the proposed neuropsychiatric SCD group, and the linear alterations of structural and functional indices were found in this group. SCD individuals with NPSs may be an essential topic for future conceptualization and research. Future work should focus on collecting biomarkers and longitudinal data to further confirm the SCD subtypes proposed here, and to better understand the underlying pathology mechanisms of SCD.

## CONFLICT OF INTEREST STATEMENT

The authors declare no conflict of interest.

## Supporting information


Appendix S1

## Data Availability

All data are available upon request from the authors.
