# Supplementary material for: Heterogeneity in subjective cognitive decline in the Sino Longitudinal Study on Cognitive Decline(SILCODE): Empirically derived subtypes, structural and functional verification
Source: CNS Neurosci Ther. 2023 Jul 20;29(12):4032–42. doi: 10.1111/cns.14327 (PMC10651943; doi:10.1111/cns.14327)
Supplement: Supplementary file 1 — Appendix S1 [file CNS-29-4032-s001.docx]

**METHODS**

*MRI data acquisition*

MRI data in the study were acquired using an integrated simultaneous 3.0 T TOF PET/MR (SIGNA PET/MR, GE Healthcare, Milwaukee, Wisconsin, USA) at the Xuanwu Hospital of Capital Medical University. Parameters for T1-weighted 3D brain structural images are as follows: Spoiled Gradient Recalled Echo (SPGR) sequence, repetition time (TR)=6.9 ms, echo time(TE)=2.98 ms, inversion time (TI)=450 ms, FOV=256×256mm^2^, matrix=256×256, slice thickness=1mm, gap=0, slice number=192, flip angle=12°,voxel size=1×1×1mm^3^. Parameters for T2-weighted 3D brain structural images are as follows: Cube T2 sequence, FOV=256 ×256 mm^2^， matrix=256 × 256，slice thickness=1mm, gap=0，slice number=192，TR=3200 ms， slice order=interleaved， voxel size=1×1×1 mm^3^. DTI data are obtained with a single-shot spin echo diffusion-weighted EPI sequence with the following parameters: FOV=224×224mm^2^, matrix=112×112, slice thickness=2mm, gap=0, slice number=70, slice order=interleaved, TR=16500 ms, TE=95.6 ms, 30 gradient directions and 5 b0 images (b=1000 s/mm^2^), voxel size=2×2×2mm^3^(the resolution of ten DTI images were 1×1×1mm^3^). A single-shot gradient-echo EPI sequence is used for rs-fMRI with the following parameters: scan duration=8min, FOV=224×224mm^2^, matrix=64×64, slice thickness=4mm, gap=1mm, slice number=28, slice order=interleaved, TR=2000 ms, TE=30 ms, flip angle=90°, voxel size=3.5×3.5×4mm^3^.

*MRI data processing*

The structural MRI images for voxel-based morphometry (VBM)^1^ analysis were pre-processed by Computational Anatomy Toolbox 12 (CAT12: https://www.nitrc.org/projects/cat/) in Statistical Parametric Mapping 12 (SPM12: www.fil.ion.ucl.ac.uk/spm) through MATLAB R2018b (MathWorks Inc., MA). The T1 images were skull-stripped and inhomogeneity-corrected; then, segmented into grey matter (GM), WM, and cerebrospinal fluid (CSF), normalized into Montreal Neurological Institute (MNI) space by Diffeomorphic Anatomical Registration using Exponentiated Lie algebra (DARTEL) registration algorithm^2^; after that, jacobian determinants modulated GM images to preserve to regional volume information; finally, the modulated GM images were isotropically smoothed by an 8 mm full-width at half-maximum (FWHM) Gaussian kernel. The volume of hippocampus, hippocampal-subfields, and cortical thickness were calculated by the minimal preprocessing pipelines for the Human Connectome Project(HCP)^3^. Cortical surface models were constructed using Freesurfer 6.0-HCP, with minor modifications to incorporate both T1 and T2 images, and parcellated by Desikan-Killiany atlas^4^. Volume of hippocampus and hippocampal-subfields were calculated by the hippo-subfields module^5^ in FreeSurfer 6.0.

The resting-state functional magnetic resonance imaging (rs-fMRI) were pre-processed through data processing & analysis for (resting-state) brain imaging (DPABI)^6^. The first 10 volumes were removed to avoid nonequilibrium magnetization effects; the remaining volumes were corrected for timing differences and head motion effects(the excluded criterion was more than 3 mm of translation or 3 degrees of rotation in any direction); then, T1 images were co-registered to the mean functional image after realignment; the transformed T1 images were then segmented into GM, WM and CSF; the corrected functional volumes were spatially normalized to MNI space and resampled to 3 mm isotropic voxels; nuisance signals (including Friston 24 head motion parameters, the WM, and CSF signals) were extracted and regressed out from the images to reduce the effects of respiratory and cardiac; the amplitude of low-frequency fluctuations (ALFF) were computed and transformed to z values; finally, the ALFF maps were smoothed with a Gaussian kernel(FWHM 4mm).

The diffusion tensor imaging (DTI) were pre-processed by a pipeline toolbox Pipeline for Analyzing braiN Diffusion imAges (PANDA)^7^. The main steps were as follows: (1) converting DICOM files to NIFIT; (2)b0 image was skull-stripped, and the brain mask was estimated (f = 0.25); (3) cropping the images (3 mm); (4) correcting for the eddy-current effects: register the diffusion-weighted images to the b0 images; (5) the diffusion tensor parameter (fractional anisotropy, FA) was calculated. FA was used for tract-based spatial statistics (TBSS) analysis^8^. All images were registered to the MNI space (FMRIB58_FA template as target image: 1×1×1 mm3), and a mean FA map was obtained by averaging the FA images from each individual in the MNI space and thinning to create a custom mean FA skeleton (skeleton cutoff = 0.2). Finally, the individual FA maps were projected onto the FA skeleton to obtain the FA skeletons of each subject, and the skeletonized FA maps were used in further analysis.

**Table S1.** Regions Showing Significant Volume Differences between SCD subtypes and NC.

| **Cluster/Peak region** | **Volume (mm^3^)** | **MNI coordinates (mm)** | | | **Peak Intensity** |
| --- | --- | --- | --- | --- | --- |
|  |  | **x** | **y** | **z** |  |
| **Cluster-derived normal vs NC** |  |  |  |  |  |
| Cerebellum_4_5_L | 2980 | -19.5 | -27 | -27 | 4.29356 |
| Cerebellum_Crus1_L | 2390 | -42 | -48 | -31.5 | 4.18723 |
| Occipital_Mid_R | 709 | 34.5 | -70.5 | 19.5 | -2.86809 |
| Cerebellum_3_R | 655 | 19.5 | -27 | -27 | 3.66734 |
| Temporal_Mid_R | 516 | 64.5 | -30 | -6 | -2.86713 |
| Temporal_Inf_R | 395 | 64.5 | -49.5 | -12 | -2.86703 |
| **Dysexecutive/mixed SCD vs NC** |  |  |  |  |  |
| Parietal_Sup_L | 2852 | -33 | -46.5 | 63 | -2.89374 |
| Cerebellum_7b_L | 2261 | -46.5 | -48 | -52.5 | 3.94521 |
| Temporal_Inf_L | 1607 | -55.5 | -16.5 | -42 | 3.8559 |
| Cerebellum_8_L | 888 | -25.5 | -42 | -51 | 3.7171 |
| **Neuropsychiatric SCD vs NC** |  |  |  |  |  |
| Rolandic_Oper_L | 1391 | -42 | -24 | 13.5 | -2.89262 |
| Calcarine_L | 1161 | -10.5 | -93 | -4.5 | -2.89548 |
| Occipital_Sup R | 1023 | 22.5 | -73.5 | 39 | -2.8931 |
| Lingual_R | 918 | 18 | -90 | -13.5 | -2.89301 |
| Temporal_Mid_R | 803 | 55.5 | -36 | -1.5 | -2.89382 |
| Cerebellum_Crus1_L | 705 | -21 | -69 | -30 | -2.89722 |
| Frontal_Mid_2_R | 574 | 33 | 39 | 33 | -2.89408 |
| Frontal_Sup_2_R | 506 | 21 | 49.5 | 46.5 | -2.89567 |
| Frontal_Med_Orb_R | 361 | 3 | 39 | -10.5 | -2.89737 |
| **Amnestic SCD vs NC** |  |  |  |  |  |
| Temporal_Mid_L | 1357 | -60 | -33 | 1.5 | -2.89539 |
| Frontal_Sup_2_L | 1188 | -13.5 | 49.5 | 30 | -2.8975 |
| Temporal_Mid_R | 1144 | 66 | -42 | 1.5 | -2.89637 |
| Frontal_Sup_2_R | 1056 | 9 | 72 | -3 | -2.89654 |
| Cerebellum_Crus1_L | 813 | -55.5 | -52.5 | -42 | 3.93763 |
| Frontal_Med_Orb_L | 800 | -3 | 49.5 | -6 | -2.89592 |
| Parietal_Sup_R | 449 | 22.5 | -66 | 46.5 | -2.8996 |
| Cerebellum_Crus1_L | 348 | -46.5 | -45 | -33 | 3.44581 |

Abbreviations: SCD, Subjective Cognitive Decline; NC, Normal Control; L, Left; R, Right.

**Table S2.** Regions Showing Significant Differences in ALFF values between SCD subtypes and NC.

| **Cluster/Peak region** | **Volume (mm^3^)** | **MNI coordinates (mm)** | | | **Peak Intensity** |
| --- | --- | --- | --- | --- | --- |
|  |  | **x** | **y** | **z** |  |
| **Cluster-derived normal vs NC** |  |  |  |  |  |
| Cerebellum_Crus1_L | 2592 | -33 | -84 | -21 | 4.10193 |
| ParaHippocampal_R | 2430 | 15 | -9 | -18 | 3.92667 |
| Frontal_Mid_2_L | 1998 | -30 | 24 | 36 | -2.87197 |
| Frontal_Sup_2_L | 1485 | -21 | 54 | 27 | -2.8869 |
| ACC_sup_L | 1242 | -12 | 30 | 27 | -2.91634 |
| Cerebellum_6_L | 1026 | -9 | -66 | -24 | -2.87395 |
| Heschl_R | 937 | 45 | -21 | 12 | 3.52323 |
| ParaHippocampal_L | 729 | -6 | -3 | -21 | 3.33514 |
| Frontal_Inf_Tri_R | 648 | 51 | 36 | 6 | -2.89515 |
| BA37_L (Fusiform gyrus_L) | 567 | -18 | -39 | -33 | -2.87646 |
| Thal_VPL_L | 567 | -15 | -24 | 0 | -2.90543 |
| Temporal_Mid_L | 567 | -48 | -51 | 21 | -2.89426 |
| **Dysexecutive/mixed SCD vs NC** |  |  |  |  |  |
| Insula_L | 702 | -33 | 12 | 15 | 4.49071 |
| Temporal_Inf_R | 675 | 42 | 3 | -42 | 3.47033 |
| **Neuropsychiatric SCD vs NC** |  |  |  |  |  |
| Putamen_R | 2835 | 30 | 9 | -9 | 4.4913 |
| Cerebellum_8_R | 1674 | 18 | -60 | -45 | 4.1481 |
| Occipital_Inf_R | 1377 | 39 | -81 | -6 | 3.68177 |
| OFCpost_R | 1080 | 27 | 21 | -21 | 4.6334 |
| Precentral_R | 918 | 48 | -15 | 45 | 3.86211 |
| Cerebellum_6_R | 648 | 15 | -72 | -24 | 4.4868 |
| Frontal_Inf_Oper_R | 540 | 33 | 9 | 27 | 4.76469 |
| **Amnestic SCD vs NC** |  |  |  |  |  |
| Occipital_Sup_L | 837 | -24 | -81 | 33 | 3.87312 |
| Frontal_Inf_Tri_L | 621 | -42 | 36 | 9 | -2.91399 |

Abbreviations: SCD, Subjective Cognitive Decline; NC, Normal Control; L, Left; R, Right.

**Table S3.** Anatomical location of significant differences in FA between the Neuropsychiatric SCD group and NC.

|  | FA |
| --- | --- |
| Total number of voxels | 28286 |
| Corpus callosum (genu, body, splenium) | √ |
| Corona radiata (anterior, superior, posterior) | L/R |
| Superior longitudinal fasciculus | L/R |
| Thalamic radiation (posterior) | L/R |
| Internal capsule (anterior, posterior, retrolenticular) | L/R |
| External capsule | L/R |
| Fornix (cres/stria) | L/R |
| Inferior fronto-occipital fasciculus | L/R |
| Cerebral peduncle | L/R |
| Uncinate fasciculus | L/R |
| Sagittal stratum | L/R |
| Cingulum | L/R |
| Tapetum | R |

Abbreviations: SCD, Subjective Cognitive Decline; NC, Normal Control; L, Left; R, Right; FA, fractional anisotropy.

**Table S4.** Differences of regional GM volumes extract from significant clusters between SCD subtypes and NC.

| **Cluster/Peak region** | **Statistical** | | | | | |
| --- | --- | --- | --- | --- | --- | --- |
|  | **1** | **2** | | **3** | | **4** |
| **Cluster-derived normal vs NC (1)** | |  | |  | |  |
| Cerebellum_4_5_L | **F=7.325; p=0.008** | | F=0.238; p=0.627 | | F=0.402; p=0.528 | F=0.007; p=0.934 |
| Cerebellum_Crus1_L | **F=6.431; p=0.013** | | F=0.283; p=0.596 | | F=0.026; p=0.873 | **F=4.478; p=0.038** |
| Occipital_Mid_R | **F=5.970; p=0.016** | | **F=9.948; p=0.002** | | F=2.076; p=0.154 | F=3.236; p=0.076 |
| Cerebellum_3_R | **F=7.485; p=0.007** | | F=0.183; p=0.670 | | F=1.352; p=0.249 | F=0.638; p=0.427 |
| Temporal_Mid_R | **F=9.305; p=0.003** | | **F=11.03; p=0.001** | | **F=7.796; p=0.007** | F=1.529; p=0.220 |
| Temporal_Inf_R | **F=9.378; p=0.003** | | F=1.836; p=0.179 | | F=1.193; p=0.278 | F=0.495; p=0.484 |
| **Dysexecutive/mixed SCD vs NC (2)** | | |  | |  |  |
| Parietal_Sup_L | F=0.226; p=0.635 | | **F=13.83; p<0.001** | | F=1.441; p=0.234 | F=1.516; p=0.222 |
| Cerebellum_7b_L | F=1.831; p=0.179 | | **F=4.246; p=0.043** | | F=1.565; p=0.215 | F=3.348; p=0.071 |
| Temporal_Inf_L | F=0.994; p=0.321 | | **F=5.085; p=0.027** | | F=0.128; p=0.722 | F=0.630; p=0.430 |
| Cerebellum_8_L | F=0.397; p=0.530 | | F=3.618; p=0.061 | | F=0.054; p=0.816 | F=0.247; p=0.621 |
| **Neuropsychiatric SCD vs NC (3)** | | |  | |  |  |
| Rolandic_Oper_L | F=0.904; p=0.344 | | F=1.209; p=0.275 | | **F=7.538; p=0.008** | F=0.761; p=0.386 |
| Calcarine_L | F=1.586; p=0.211 | | F=3.517; p=0.065 | | **F=9.518; p=0.003** | F=0.693; p=0.408 |
| Occipital_Sup_R | F=2.660; p=0.106 | | **F=4.391; p=0.040** | | **F=10.18; p=0.002** | F=0.907; p=0.344 |
| Lingual_R | F=1.090; p=0.299 | | F=2.145; p=0.147 | | **F=10.23; p=0.002** | F=0.985; p=0.324 |
| Temporal_Mid_R | **F=6.610; p=0.012** | | **F=6.721; p=0.011** | | **F=8.630; p=0.004** | **F=6.841; p=0.011** |
| Cerebellum_Crus1_L | F=0.505; p=0.479 | | F=2.042; p=0.157 | | **F=6.783; p=0.011** | F=0.156; p=0.694 |
| Frontal_Mid_2_R | F=0.372; p=0.543 | | **F=5.468; p=0.022** | | **F=9.160; p=0.003** | F=0.500; p=0.482 |
| Frontal_Sup_2_R | F=1.001; p=0.319 | | **F=5.326; p=0.024** | | **F=10.45; p=0.002** | F=0.681; p=0.412 |
| Frontal_Med_Orb_R | F=1.169; p=0.282 | | F=3.838; p=0.054 | | **F=8.240; p=0.005** | F=2.748; p=0.102 |
| **Amnestic SCD vs NC (4)** | | |  | |  |  |
| Temporal_Mid_L | F=2.112; p=0.149 | | F=1.131; p=0.291 | | **F=5.850; p=0.018** | **F=7.912; p=0.006** |
| Frontal_Sup_2_L | F=0.024; p=0.876 | | F=3.625; p=0.061 | | F=1.512; p=0.223 | **F=7.367; p=0.008** |
| Temporal_Mid_R | **F=8.223; p=0.005** | | **F=5.440; p=0.022** | | F=2.634; p=0.109 | **F=5.367; p=0.023** |
| Frontal_Sup_2_R | F=0.577; p=0.449 | | F=1.299; p=0.258 | | F=2.549; p=0.115 | **F=6.119; p=0.016** |
| Cerebellum_Crus1_L | F=3.142; p=0.079 | | F=0.185; p=0.668 | | F=0.235; p=0.629 | **F=12.51; p=0.001** |
| Frontal_Med_Orb_L | F=1.281; p=0.260 | | F=2.600; p=0.111 | | **F=5.136; p=0.026** | **F=5.628; p=0.020** |
| Parietal_Sup_R | **F=4.852; p=0.030** | | F=0.195; p=0.660 | | F=2.374; p=0.128 | **F=8.086; p=0.006** |
| Cerebellum_Crus1_L | **F=4.279; p=0.041** | | F=0.057; p=0.812 | | F=0.018; p=0.895 | **F=7.828; p=0.007** |

Abbreviations: SCD, Subjective Cognitive Decline; NC, Normal Control; L, Left; R, Right.

**Table S5.** Differences of regional ALFF values extract from significant clusters between SCD subtypes and NC.

| **Cluster/Peak region** | **Statistical** | | | |
| --- | --- | --- | --- | --- |
|  | **1** | **2** | **3** | **4** |
| **Cluster-derived normal vs NC (1)** | |  |  |  |
| Cerebellum_Crus1_L | **F=11.8;p=0.0002** | F=0.400; p=0.529 | F=2.859; p=0.095 | **F=5.225;p=0.025** |
| ParaHippocampal_R | **F=16.8;p=0.00008** | F=2.791; p=0.099 | **F=6.745; p=0.011** | **F=5.03; p=0.028** |
| Frontal_Mid_2_L | **F=6.766; p=0.011** | F=0.816; p=0.369 | F=0.681; p=0.412 | **F=5.846;p=0.018** |
| Frontal_Sup_2_L | **F=7.999; p=0.006** | **F=8.040;p=0.006** | **F=5.213; p=0.025** | F=0.618;p=0.434 |
| ACC_sup_L | **F=9.053; p=0.003** | F=0.274; p=0.602 | F=0.885;p=0.350 | F=0.905;p=0.345 |
| Cerebellum_6_L | **F=9.408; p=0.003** | F=0.001; p=0.974 | F=1.309; p=0.256 | F=3.167; p=0.079 |
| Heschl_R | **F=11.40; p=0.001** | F=0.299; p=0.586 | F=0.065; p=0.800 | F=3.511; p=0.065 |
| ParaHippocampal_L | **F=10.11; p=0.002** | F=1.732; p=0.192 | F=3.153; p=0.080 | F=0.670; p=0.416 |
| Frontal_Inf_Tri_R | **F=8.200; p=0.005** | **F=5.647; p=0.020** | **F=5.946; p=0.017** | F=1.300; p=0.258 |
| BA_37_L | **F=8.619; p=0.004** | F=0.134 p=0.716 | F=1.976; p=0.164 | F=0.107;p=0.745 |
| Thal_VPL_L | **F=10.66; p=0.001** | F=0.139; p=0.711 | F=0.196; p=0.660 | F=0.342;p=0.561 |
| Temporal_Mid_L | **F=6.499; p=0.012** | F=0.224; p=0.637 | F=1.116; p=0.294 | F=0.959;p=0.331 |
| **Dysexecutive/mixed SCD vs NC (2)** | |  |  |  |
| Insula_L | F=1.320; p=0.253 | **F=17.5;p=0.00008** | F=1.484; p=0.227 | F=0.532; p=0.468 |
| Temporal_Inf_R | **F=5.456; p=0.021** | **F=11.71; p=0.001** | **F=5.611; p=0.020** | F=1.145; p=0.288 |
| **Neuropsychiatric SCD vs NC (3)** | |  |  |  |
| Putamen_R | F=0.579; p=0.111 | F=0.424; p=0.517 | **F=20.09;p=0.00003** | F=0.010; p=0.919 |
| Cerebellum_8_R | F=2.218; p=0.139 | F=0.099; p=0.753 | **F=12.02;p=0.001** | F=0.017; p=0.897 |
| Occipital_Inf_R | **F=9.053; p=0.003** | F=2.935; p=0.091 | **F=10.919; p=0.001** | F=0.625; p=0.432 |
| OFCpost_R | F=2.419; p=0.123 | F=2.228 p=0.140 | **F=17.15;p=0.00009** | F=0.355; p=0.553 |
| Precentral_R | F=0.988; p=0.323 | F=0.763; p=0.385 | **F=13.86; p=0.0004** | F=2.108; p=0.151 |
| Cerebellum_6_R | F=0.001; p=0.970 | F=0.072; p=0.789 | **F=11.433;p=0.001** | F=1.907; p=0.172 |
| Frontal_Inf_Oper_R | F=2.299; p=0.133 | **F=7.255; p=0.009** | **F=21.09;p=0.00002** | F=1.046; p=0.310 |
| **Amnestic SCD vs NC (4)** | |  |  |  |
| Occipital_Sup_L | F=0.552; p=0.459 | **F=6.421; p=0.013** | **F=6.371; p=0.014** | **F=12.2; p=0.001** |
| Frontal_Inf_Tri_L | **F=5.308; p=0.023** | **F=5.457; p=0.022** | **F=9.672; p=0.003** | **F=10.1; p=0.002** |

Abbreviations: SCD, Subjective Cognitive Decline; NC, Normal Control; L, Left; R, Right.


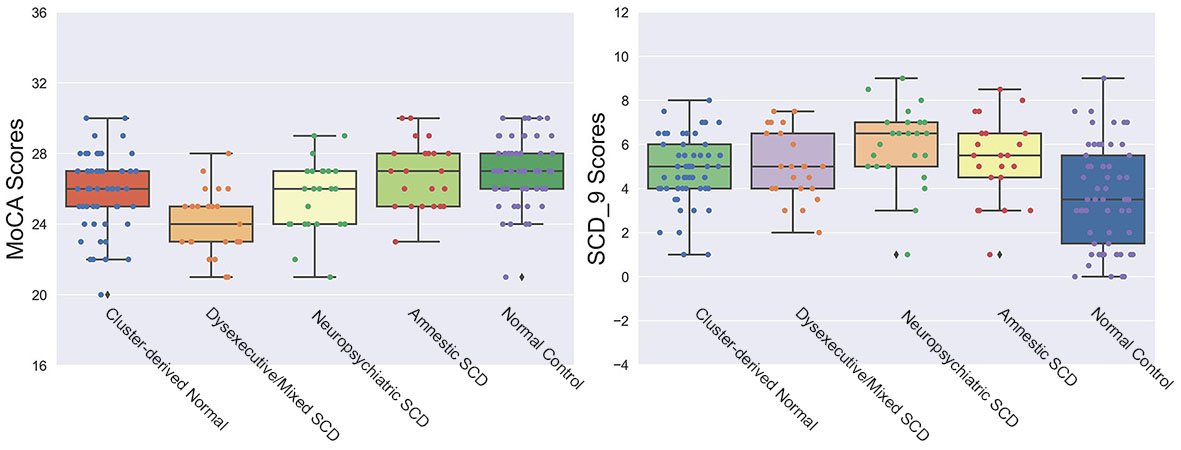


**Fig. S1.** Box-and-whisker plots of the neuropsychological data in the four subgroups and normal control group


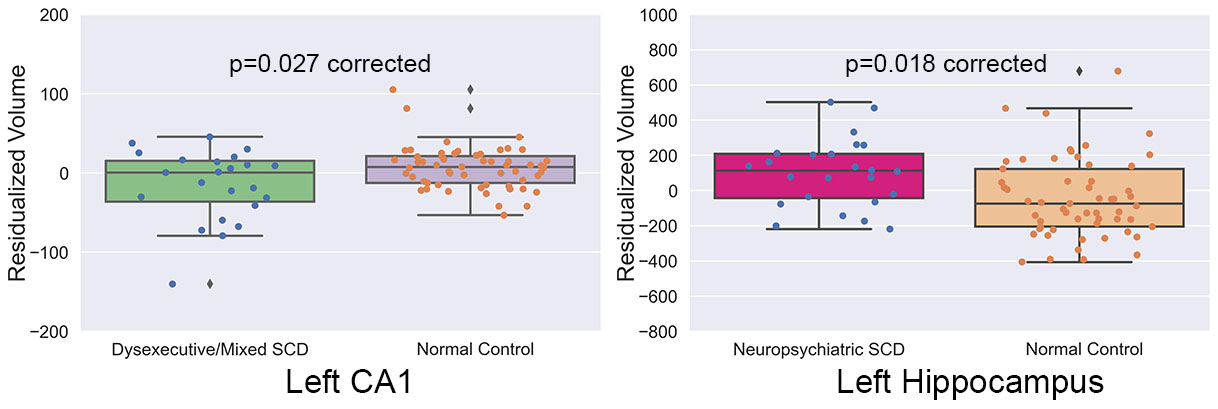


**Fig. S2.** Box-and-whisker plots of the between-group differences in the hippocampus and hippocampal-subfields volumes for the subgroups


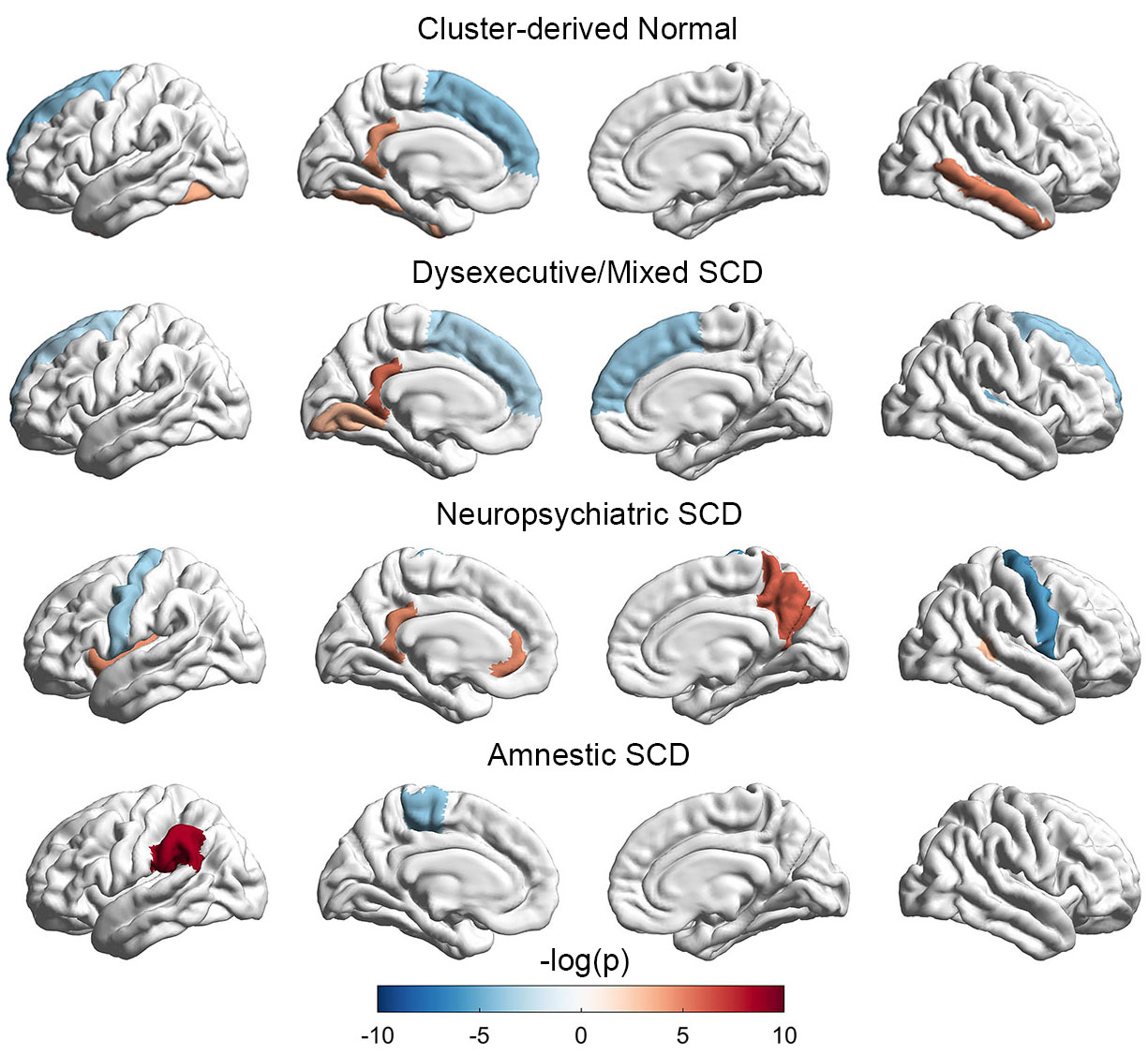


**Fig. S3.** Regional cortical thickness on the left and right lateral and medial pial surfaces for each cluster-derived group relative to the normal control group. The cortical thickness of the left supramarginal was thinner in the amnestic SCD group than in the NC group(p=0.0128) after Bonferroni correction.

**REFERENCES**

1. Ashburner J, Friston KJ. Voxel-based morphometry--the methods. NeuroImage 2000;11:805-821.

2. Ashburner J. A fast diffeomorphic image registration algorithm. NeuroImage 2007;38:95-113.

3. Glasser MF, Sotiropoulos SN, Wilson JA, et al. The minimal preprocessing pipelines for the Human Connectome Project. NeuroImage 2013;80:105-124.

4. Desikan RS, Ségonne F, Fischl B, et al. An automated labeling system for subdividing the human cerebral cortex on MRI scans into gyral based regions of interest. NeuroImage 2006;31:968-980.

5. Iglesias JE, Augustinack JC, Nguyen K, et al. A computational atlas of the hippocampal formation using ex vivo, ultra-high resolution MRI: Application to adaptive segmentation of in vivo MRI. NeuroImage 2015;115:117-137.

6. Yan CG, Wang XD, Zuo XN, Zang YF. DPABI: Data Processing & Analysis for (Resting-State) Brain Imaging. Neuroinformatics 2016;14:339-351.

7. Cui Z, Zhong S, Xu P, He Y, Gong G. PANDA: a pipeline toolbox for analyzing brain diffusion images. Frontiers in human neuroscience 2013;7:42.

8. Smith SM, Jenkinson M, Johansen-Berg H, et al. Tract-based spatial statistics: voxelwise analysis of multi-subject diffusion data. NeuroImage 2006;31:1487-1505.
